# Transactional e-health literacy and its association with e-health services use in Polish adults: a cross-sectional study

**DOI:** 10.3389/fdgth.2024.1458650

**Published:** 2024-11-22

**Authors:** Paulina Smoła, Urszula Zwierczyk, Mariusz Duplaga

**Affiliations:** Department of Health Promotion and e-Health, Institute of Public Health, Faculty of Health Sciences, Jagiellonian University Medical College, Kraków, Poland

**Keywords:** transactional e-health literacy, e-health, remote physician advice, cultural adaptation, exploratory factor analysis, confirmatory factor analysis

## Abstract

**Introduction:**

The transactional model of e-health literacy addresses not only the skills needed for handling online health-related information but also the capacity to communicate regarding health issues on the Internet. It also emphasizes a critical component of e-health literacy: enabling appraisal and selection of information adequate to individual needs. Our study aimed to culturally adapt the instrument assessingTransactional e-Health Literacy (TeHL) and examine the association between TeHL and the use of e-health services by Polish adult Internet users.

**Methods:**

The analysis was conducted on data from an online survey among 1,661 respondents. After cultural adaptation and piloting of the Polish version of the instrument measuring TeHL, exploratory and confirmatory factor analyses were performed on two samples obtained by random splitting of the original data set. The roles of TeHL categories in the use of several types of e-health services were assessed with multivariable logistic regression models.

**Results:**

We have found that the four-factor model of the Polish version of the TeHL instrument, consisting of 17 items, obtained after excluding item 13, shows the best fit to the measurement data (NFI = 0.950, RFI 0.938, TLI = 0.951, CFI = 0.960, GFI = 0.932, RMSEA = 0.066). Regression modeling revealed that Functional e-health literacy is a significant positive predictor of the use of remote physician advice, the Internet Patient Account Portal, portals providing general health-related information, and websites allowing for checking laboratory test results. Communicative eHL was significantly negatively related to the use of general health-related information portals and positively related to the use of portals offering paid medical advice. Critical e-health literacy was a significant negative predictor of the use of remote physician advice and the laboratory test results websites but a positive predictor of using portals offering paid medical advice and websites offering easy access to e-prescriptions. Finally, Translational e-health literacy was significantly positively associated with the use of the Internet Patient Account Portal, general health-related information portals, and laboratory test results websites.

**Discussion:**

Polish version of the instrument assessing TeHL is a tool of confirmed validity that can be used for e-health research in Poland. The relationships between four types of TeHL and the use of concrete e-health solutions show a complex pattern requiring further evaluation.

## 1 Introduction

The COVID-19 pandemic resulted in considerable growth in the use of e-health services in many countries (1). Healthcare providers realized that such services enable maintaining social distance and avoiding the risk of being infected for both interacting parties. Even in countries with relatively low levels of usage of e-health services, as in Poland, the pandemic led to a spectacular increase in their use due to the quick introduction of reimbursement schemes (2).

From March to June 2020, about 80% of visits to general practitioners were conducted remotely, mainly by telephone (2). Consecutive studies also confirmed the common use of other e-health services, fortunately introduced before the pandemic, e.g., e-prescriptions (3). Although in the following years, the Ministry of Health narrowed the scope of medical problems that could be addressed by teleadvice, the usage of this option in primary and specialist ambulatory care has remained high (4).

One can also observe an increase in the popularity of the Internet Patient Account Portal (IPAP) maintained by the government to provide citizens with information about delivered health services (3). According to a study conducted in October 2020, 17% of adult Internet users had accessed the the Internet Patient Account Portal at least once. After the introduction of COVID-19 vaccinations, the Portal was commonly used for retrieving the certificates of vaccination required by many countries during border controls (3). The survey carried out in June 2023 revealed that the use of the Portal by Polish citizens had increased to 43% (3).

Our earlier studies showed that the determinants of satisfaction with using and the readiness to use e-health services included, apart from health (HL) and e-health literacy (eHL), the aim of remote physician advice and the technical means used for obtaining it (5). In these studies, we assessed the eHL of respondents on the e-Health Literacy Scale (eHEALS) developed by Norman and Skinner in 2006 (6) and adapted to Polish in 2019 (7).

The concept of eHL was introduced by Norman and Skinner to reflect the set of skills needed to efficiently handle health information available online (6). They developed the model of eHL assuming that there are several core literacy types forming the background for eHL. The model, depicted as lily petals (and called the Lily Model), encompassed traditional literacy and numeracy, computer literacy, media, science, information, and, finally, health literacy. According to Norman and Skinner, the Lily Model consisted of two components: analytical and context-specific. The first component was mainly dependent on media and information literacy. The latter was supposed to rely on situation-specific skills and covered computer, scientific, and health literacies. In general, eHL was perceived as a set of skills empowering individuals in making health-related decisions based on e-health resources.

Following their concept of the six core literacies, Norman and Skinner developed an instrument to measure the level of eHL in the population, the eHealth Literacy Scale (eHEALS) (8). The scale was designed as a self-reported tool allowing respondents to assess their skills related to managing health information. The tool quickly became popular and commonly used in e-health research. The eHEALS was adapted to many languages and validated among various groups of respondents (7, 9).

Shortly after the introduction of the eHEALS, some authors commented on the shortcomings of its theoretical background. Even Norman mentioned in 2011 that the concept of eHL proposed in 2006 may require adjustment due to the growth of social media and new trends in the roles assumed by Internet users, especially concerning the provision of their own content and using retrieved health information for solving problems (10). Other authors questioned the ability of the eHEALS to measure all six core literacies included in the Lily Model (11). Some voices were even raised to contest the validity of the eHEALS (12). According to the initial validation study of Norman and Skinner, the eHEALS had a single-factor structure. This seemed counterintuitive, taking into consideration their definition of eHL as addressing four main skills: seeking, finding, understanding, and appraising the health information available from electronic sources (6). However, the majority of validation studies confirmed the single-factor structure of the scale (7, 12–14). Only a few studies reported that the scale had a two-factor (15, 16) or even the three-factor structure (17).

In search of a tool that would correspond with the quickly changing landscape of e-health and the skills needed to navigate the digital health domain, new instruments with multidimensional structures were proposed (18–20). Paige et al. developed an instrument based on the Transactional Model of e-Health Literacy (21). Following the results of a systematic review, they reported an incongruity between operational eHL, as addressed in existing definitions, and the literacies included in the available models and measures. They also emphasized the insufficient role of communication in the concepts of eHL, even though transactional capabilities are essential for the use of e-health (21). According to their model, the set of skills forming eHL is operationalized through four competencies: functional, communicative, critical, and translational. Functional skills enable finding and understanding health information from electronic sources, communicative skills are responsible for the exchange of information, critical for its assessment, and finally, translational for decision making. The instrument developed for the measurement of the Transactional e-Health Literacy (TeHL) based on the model of Paige et al. was confirmed to have a four-factor structure. Four distinguished subscales corresponded with the above-mentioned operational skills, with 4–5 items in each subscale (20).

Our study aimed to culturally adapt and validate the instrument for the assessment of TeHL. We also analyzed the relationship between transactional TeHL and the use of e-health services by adult Internet users in Poland. We developed multivariable logistic regression models of the use of selected e-health services, adjusting the effect of TeHL for sociodemographic factors. The services were proposed based the information from earlier surveys showing their use in society (5). We have decided to develop models for six dichotomous variables reflecting the use of remote physician advice, the governmental Internet Patient Account Portal, portals with general health-related information, websites allowing users to check laboratory test results, websites providing paid medical advice to customers, and finally, websites offering e-prescriptions in the preceding 12 months. Taking into consideration the earlier findings of Paige et al. of positive correlations between scores derived from the four subscales of the instrument for assessing of TeHL, we hypothesized that the use of the above-listed e-health services would be higher among respondents with higher levels of the subscores established for the Polish version of the instrument.

## 2 Material and methods

### 2.1 Survey

In this study, we analyzed the data originating from a computer-assisted web-based interviewing (CAWI) survey among a representative sample of 1,661 adult Internet users aged 18–75. The study sample was adjusted according to age, education, gender, place of residence, and NUTS1 region to comply with the characteristics of adult Internet users in Poland for 2022, provided by Statistics Poland, the national statistical bureau (22). Assuming a confidence level of 0.95 and a fraction of 0.5, the sampling error was 2.4% [taking into consideration the population of Internet users aged 18–74 of about 26,370,000 in 2022 (22)]. The survey was carried out in July 2023 by Ogólnopolski Panel Badawczy, a company specializing in online studies of public opinion. The respondents invited to the survey were selected from the Ariadna Internet Panel, gathering 150,000 active participants and maintained by the company (23).

The study was approved by the Bioethical Committee of Jagiellonian University (Decision No 1072.6120.99.2020 from April 23, 2020, with amendments). Respondents invited to participate in the survey received information about the study's aims and expected outcomes. They were required to provide their informed consent to join the survey before accessing the online questionnaire.

### 2.2 Questionnaire

The questionnaire applied in the survey consisted of 107 individual items, including the following instruments: the 6-item European Health Literacy Survey Questionnaire (HLS-EU-Q6) (24), the 10-item e-Health Literacy Scale (eHEALS) (7, 8), the 11-item Technology Anxiety Scale (25), the 7-item eHealth Readiness Scale (26), the 18-item TeHLI (20), the Telehealth Usability Questionnaire (27) adapted and applied to assess respondents' attitudes to remote physician advice services, and finally, the 10-item System Usability Scale (28) applied to assess respondents' opinions about the Internet Patient Account Portal. Apart from these tools, the questionnaire encompassed a set of items asking about the use of e-health services, health status, and socio-demographic characteristics.

In this study, responses to the Polish version of the instrument for the measurement of TeHL and the eHEALS and items asking about sociodemographic features were analyzed. The eHEALS consists of eight items with five response options based on the Likert scale from “I decidedly do not agree” to “I decidedly agree.” The total eHL score is calculated as a sum of responses converted to numerical values (from 1 to 5).

### 2.3 Cultural adaptation of the TeHLI

The authors received consent to culturally adapt the instrument for the measurement of TeHL to Polish from Samantha Paige (personal communication from March 6, 2023). We applied the World Health Organization guidelines for transcultural adaptation of research tools (29). Two forward translations of TeHLI were prepared by native-Polish-speaking persons with professional backgrounds in medicine or public health and digital health. The main rule guiding the translation was developing a conceptual equivalent rather than a word-for-word translation. The translators were instructed to use expressions relevant to the Polish cultural context. Professional, technical, medical, and scientific language was avoided.

Two versions of translations of the instrument to Polish were assessed and discussed by the expert panel consisting of five persons with backgrounds in medicine, nursing, public health, computer science, and sociology. The panel was asked to provide their opinions about the developed translations. The final version of the translation was established by consensus.

In the next stage, two translators, with English as their native language, developed a backward translation of the agreed Polish version of the tool. Backward translators were independent of the research team and were not professionally associated with medicine, public health, or computer science. They were not acquainted with the English version of the tool. Two English versions of the instrument, original and back-translated, were assessed for the parts that could be distorted in translation. The terms essential for the domain of the instrument were also critically analyzed.

Piloting, including cognitive interviewing of the agreed Polish version of the instrument, was conducted among a group of 12 respondents. The pilot group consisted of 58% women, 50% inhabitants of large cities, and 25% inhabitants of rural or urban areas with population <20,000; 42% of participants had achieved a university master's level education. Participants of the piloting phase used the Internet at least 3–5 h weekly and social media at least 15–30 min daily. They received paper forms with additional fields for all items of the instrument to provide feedback critical for cognitive interviewing. They were supposed to explain what they were thinking when they answered consecutive items, why they had selected a given response, and whether they understood all the terms and expressions used in the instrument. They were also asked to indicate the words, expressions, or parts of items that were not fully understandable. The lack of feedback or responses, which were not fully clear were individually discussed with the participants by research team members.

After concluding the pilot phase, participants' responses were discussed and final amendments were introduced to the instrument. Two participants indicated they did not know what the Polish equivalent of “basic health information” means. As this expression was used in several items (Items 1, 2, 4), examples were added to the first item with this expression (“e.g., information related to healthy nutrition or physical activity”). There were four participants who did not understand the expression “health needs” (Item 3). The final version of Item 3 was also amended with examples formulated as “e.g., needs related to screening tests or healthy lifestyle”). The Polish translation of Item 5 used for the English expression, “I can achieve my health information goals…” was not fully clear for another four participants. Following the feedback from piloting, item 5 was simplified in Polish and more natural language was used in it. Some minor amendments were introduced to Items 9 and 11, relying on the substitution of vocabulary with more common words (e.g., “untrue” instead of “false”). Both the original English and the adapted Polish versions of TeHLI are included in the Supplementary Table S1.

### 2.4 Statistical analysis

The statistical analysis was performed with the IBM SPSS v.29 and IBM SPSS Amos 29 (IBM Corp. Armonk, NY, USA) programs. Absolute and relative frequencies were obtained for categorical variables, means, and standard deviations (SD) for continuous numerical variables.

The internal consistency of the Polish version of the instrument was analyzed based on the Cronbach *α* coefficient. It was assumed that a coefficient between 0.7 and 0.9 indicates good, and ≥0.9 indicates excellent internal consistency. A Guttman split-half coefficient of at least 0.8 was assumed to reveal sufficient internal consistency of the scale. The floor and ceiling effects were assessed based on the percentage of respondents who reached minimum and maximum levels for subscores established for factors resulting from EFA.

The temporal stability of the instrument was assessed with a test-retest procedure. The group of 100 respondents from the first wave of the survey filled out the questionnaire once again two weeks later. Based on the data from the two waves of the survey in this group, mean and single-item interclass correlation coefficients (ICC) were assessed in the two-way mixed model. The first coefficient was applied to analyze the stability averaged across all respondents, and the second was for the assessment of the stability of an idealized single rater. Mean ICC values <0.40 correspond with poor, 0.40–0.59 fair, 0.60–0.74 good and 0.75–1.00 excellent stability.

The adequacy of the sample size to the number of items in the scale was assessed via the Kaiser-Meyer-Olkin test. It was expected that a test result above 0.7 would confirm the adequacy of the sample size (30). The factorability of the data was analyzed with Barlett's test of sphericity. Multicollinearity was assessed based on the correlation. It was expected that the correlation between the two items should not be greater than 0.8 (31).

Hypotheses testing was applied to assess the construct validity of the scale. The correlations between the eHEALS score and the scores calculated for the TeHLI were analyzed. Furthermore, the relationship between the scores derived from the TeHLI and the use of e-health services was analyzed based on univariate regression models.

### 2.5 Exploratory factor analysis

Exploratory (EFA) and confirmatory (CFA) factor analyses were conducted on two datasets procured through random splitting of the initial dataset obtained from the survey. The EFA was preceded by the assessment of the communalities values; the threshold value for the communality was 0.2 (32).

The EFA, based on the maximum likelihood method and direct oblimin rotation, was performed in two variants: enforcing the four-factor model as reported for an original version of the instrument (20) and allowing for the automatic establishment of the factor structure. Direct oblimin rotation technique decreases the cross products of loadings to simplify factors and permit the factors to be correlated (33).

In the EFA variant without an enforced number of factors, only factors with an eigenvalue of at least 1.0 were preserved following the Kaiser criterion. We assumed that the extracted factors should be responsible for at least 50% of the total variance (34). Pattern and structure matrices were applied to assess factor loadings. It was also expected that loadings would be greater than 0.4 (31, 35). Furthermore, cross-loadings were checked; a recommended ratio of loadings was assumed to be <0.75% in the pattern matrix. It was also assumed that the extracted factor should contain at least three items adhering to the mentioned criteria.

### 2.6 Confirmatory factor analysis

CFA was used to check the factor structure of the Polish version of the instrument for TeHL obtained with the EFA. The CFA was conducted on the second dataset acquired by randomly splitting of initial survey data. The maximum likelihood method was used for the estimation of the model during the CFA. The fits of three models were analyzed, including the four-factor model proposed for the original English version of the instrument and variants of the model received after the exclusion of selected items based on the criteria applied to the loadings' threshold described earlier for the EFA.

Several fit indices were applied to analyze the goodness-of-fit of the models. They included the chi-squared-to-degrees-of-freedom ratio (CDFR), the goodness-of-fit index (GFI), the adjusted GFI according to degrees of freedom (AGFI), the Tucker and Lewis Index (TLI), the normed fit index (NFI), the comparative fit index (CFI), and the root-means-square error of approximation (RMSEA). The interpretation of the model fit was performed based on the recommendations indicated in the available literature (36, 37). We assumed that the acceptable fit level for CDFR should be <5.0 and the good fit level <2.0. The acceptable level for NFI, RFI, and TLI was assumed to be >0.90, for GFI at least 0.85, and for AGFI at least 0.80. Finally, it was assumed that a RMSEA <0.05 shows good, and from 0.05 to 0.08 acceptable fit. It was also expected that at least five indices should reach reference levels to assume that there is an acceptable goodness-of-fit of the data to the factor structure.

### 2.7 Logistic regression modeling

The associations of TeHL with the use of selected e-health services were analyzed with multivariable regression models, adjusting for sociodemographic variables. The scores based on the subscales distinguished in the model of the TeHLI were introduced to the regression models as independent variables. Before models were developed, multicollinearity was checked. None of the variables met the criteria of multicollinearity (tolerance <0.25, VIF > 4). The Hosmer-Lemeshow test and Nagelkerke R2 were calculated for the developed regression models. Odds ratios and 95% confidence intervals, as well as *p* values, were reported to show the effect of the independent variables. In all analyses, a *p*-value <0.05 was treated as significant.

## 3 Results

### 3.1 Characteristics of the study sample

The characteristics of the study sample and the samples obtained after the random splitting of the initial data set are provided in Table 1. It shows that the socio-demographic characteristics of subsets used for the EFA and the CFA do not differ significantly.

**Table 1:** Characteristics of the study samples (*n* = 1,661) and subsamples (*n* = 796 for EFA, and *n* = 865 for CFA).

		All subjects % (*n*)	EFA subgroup	CFA subgroup
Gender	Female	52.9 (879)	53.0 (422)	52.8 (457)
	Male	47.1 (782)	47.0 (374)	47.2 (408)
Place of residence	Rural	38.2 (634)	39.7 (316)	36.8 (318)
	Urban <20,000	11.9 (197)	11.8 (94)	11.9 (103)
	Urban 20,000 to <100,000	20.9 (347)	20.2 (161)	21.5 (186)
	Urban 100,000 to <200,000	8.4 (139)	8.5 (68)	8.2 (71)
	Urban 200,000 to <500,000	8.4 (139)	7.9 (63)	8.8 (76)
	Urban ≥500,000	12.3 (205)	11.8 (94)	12.8 (111)
Education	Lower than secondary	22.0 (365)	20.6 (164)	23.2 (201)
	Secondary	40.0 (6,640	40.6 (323)	39.4 (341)
	Post-sec. non-University	7.5 (124)	7.7 (61)	7.3 (63)
	University Bachelor's	8.5 (142)	7.8 (62)	9.2 (80)
	University Master's	22.0 (366)	23.4 (186)	20.8 (180)
Marital status	Single	21.6 (358)	20.5 (163)	22.5 (195)
	Married	52.1 (866)	54.0 (430)	50.4 (436)
	In partnership	14.3 (238)	13.4 (107)	15.1 (131)
	Widowed	4.7 (78)	3.9 (31)	5.4 (47)
	Divorced or separated	7.3 (121)	8.1 (65)	6.5 (56)
Vocational status	Employee	51.7 (858)	51.8 (412)	51.6 (446)
	Self-employed or farmer	9.5 (158)	10.1 (80)	9.1 (78)
	Retired or on disability pension	23.5 (391)	24.6 (196)	22.6 (195)
	University or high school student	3.9 (65)	3.1 (25)	4.7 (40)
	Unemployed or other	11.4 (189)	10.4 (83)	12.3 (108)
Net monthly income	<2,001 PLN	23.2 (386)	21.5 (171)	24.9 (215)
	2,001–3,000 PLN	22.9 (381)	24.9 (198)	21.2 (183)
	3,001–5,000 PLN	24.4 (406)	24.2 (193)	24.6 (213)
	>5,000 PLN	12.5 (208)	12.3 (98)	12.7 (110)
	Not revealed	16.9 (280)	17.1 (136)	16.6 (144)

### 3.2 Item analysis of the initial Polish version of the adapted instrument for assessing TeHL

The means (standard deviation) of the items included in the initial 18-item Polish version of the instrument spanned from 3.09 (1.04) for item 6 to 4.02 (0.68) for item 18 (Table 2). The Item-Factor correlation coefficient ranged from 0.56–0.77. Finally, initial commonalities ranged from 0.56–0.78.

**Table 2:** Item characteristics of the initial 18-item Polish version of the TeHLI.

Item	Item description	Means (SD)	Item-factor correlation coefficient	Cronbach's *α* if item deleted	Initial commonalities
1		3.70 (0.77)	0.64	0.953	0.56
2		3.93 (0.75)	0.71	0.952	0.72
3		3.67 (0.86)	0.75	0.951	0.70
4		3.82 (0.81)	0.76	0.951	0.74
5		3.54 (0.86)	0.76	0.951	0.75
6		3.09 (1.04)	0.69	0.952	0.78
7		3.29 (0.94)	0.77	0.951	0.72
8		3.16 (1.00)	0.74	0.951	0.77
9		3.18 (0.95)	0.76	0.951	0.77
10		3.24 (0.93)	0.73	0.951	0.78
11		3.33 (0.88)	0.70	0.952	0.78
12		3.32 (0.89)	0.71	0.952	0.72
13		3.77 (0.74)	0.72	0.952	0.66
14		3.30 (0.90)	0.72	0.952	0.76
15		3.76 (0.80)	0.74	0.951	0.70
16		3.65 (0.85)	0.72	0.952	0.64
17		3.73 (0.77)	0.75	0.951	0.76
18		4.02 (0.68)	0.56	0.954	0.64

### 3.3 Exploratory factor analysis for the Pl-TeHLI

The Kaiser-Meyer-Olkin test result of 0.952 revealed that the sample size was adequate to conduct the EFA. The correlation matrix's factorability was confirmed with Barlett's test (*χ*^2^ = 11, 612.91, *p* < 0.001). The communality scores ranged from 0.56 to 0.78.

In the variant EFA with an enforced four-factor model, initial eigenvalues calculated for the four factors were 10.22, 1.56, 1.14, and 0.80; and 9.92, 1.26, 0.86, and 0.48 after rotation (Supplementary Table S2). They were responsible for 76.03% of the variance before and for 69.50% of the variance after rotation. Factors distinguished in the four-factor model corresponded with the Functional, Communicative, Critical, and Translational factors described by Paige et al. for the English version of the instrument (20). However, our analysis showed that item 5 (“I can achieve my health information goals on the Internet while helping other users achieve theirs”) should be included in Factor 1 (functional) and not in Factor 2 (communicative). We decided that its phrasing did allow for such a shift.

The minimum factor loading after rotation was 0.38 for item 13 to factor 4 (translational). The pattern matrix with factors’ loadings resulting from the EFA based on the maximum likelihood method and direct oblimin rotation is provided in Supplementary Table S3. The cross-loading of item 13 between factor 3 (critical) and factor 4 (communicative) was 0.85. Taking into consideration the loading for this item below the expected level of 0.4 and, additionally, significant cross-loading between two factors, we have decided to exclude it from the model. The pattern matrix generated with the EFA without item 13 revealed stable loading of all items and no significant cross-loadings.

We have also analyzed the factor model generated without prior enforcement of the factors’ numbers using the criteria described in the Methods section. The automatically yielded model consisted of three factors with initial eigenvalues above 1.0 in agreement with the Kaiser criterion. Those three factors explained 71.81% of the variance. The eigenvalues of these three factors after rotation were 10.22, 1.56, and 1.14. Item 5 was excluded from the three-factor model due to significant cross-loading between two factors. Factor 1 in this model combined items originally assigned to the “Functional” factor (items 1–4), item 13 from the “Critical” factor, and items 15–18 from the “Translational” factor. Two other factors roughly corresponded with factors distinguished in the original version of the instrument: factor 2 (“Communicative”) encompassed items 6–9, and factor 3 (“Critical”) items 10–12 and 14.

### 3.4 Confirmatory factor analysis

We have performed a CFA for three models: the four-factor model corresponding with the original model reported by Paige et al. for the English version of the instrument (20), the four-factor model after the exclusion of item 13, and the three-factor model after the exclusion of item 5. The fit indices for these three variants of the TeHLI model are shown in Table 3.

**Table 3:** The fitting results of the one-factor model of the Polish version of the TeHLI.

Fit indices	Threshold levels of indices	Fitting of the four-factor model after exclusion of item 13	Fitting of the original four-factor model	Fitting of the three-factor model after exclusion of item 5
CMIN/DF	Recommended <2.0 (*p* > 0.05), acceptable <5.0	4.697 (*p* < 0.001)	6.746 (*p* < 0.001)	5.59 (*p* < 0.001)
NFI	Acceptable: ≥0.90 to <0.95, good: ≥0.95	0.950	0.924	0.938
RFI	Acceptable: ≥0.90 to <0.95, good: ≥0.95	0.938	0.907	0.926
TLI	Acceptable: 0.90–0.95, good: >0.95	0.951	0.920	0.938
CFI	Acceptable: 0.90–0.95, good: ≥0.95	0.960	0.934	0.949
GFI	Acceptable: ≥0.85 to <0.95, good: ≥0.95	0.932	0.895	0.920
AGFI	Acceptable: ≥0.80 to <0.95, good: ≥0.95	0.907	0.858	0.892
RMSEA	Acceptable: <0.08 to 0.05, good: <0.05	0.065 (0.060–0.071)	0.082 (0.076–0.087)	0.073 (0.067–0.078)

**Figure 1:**
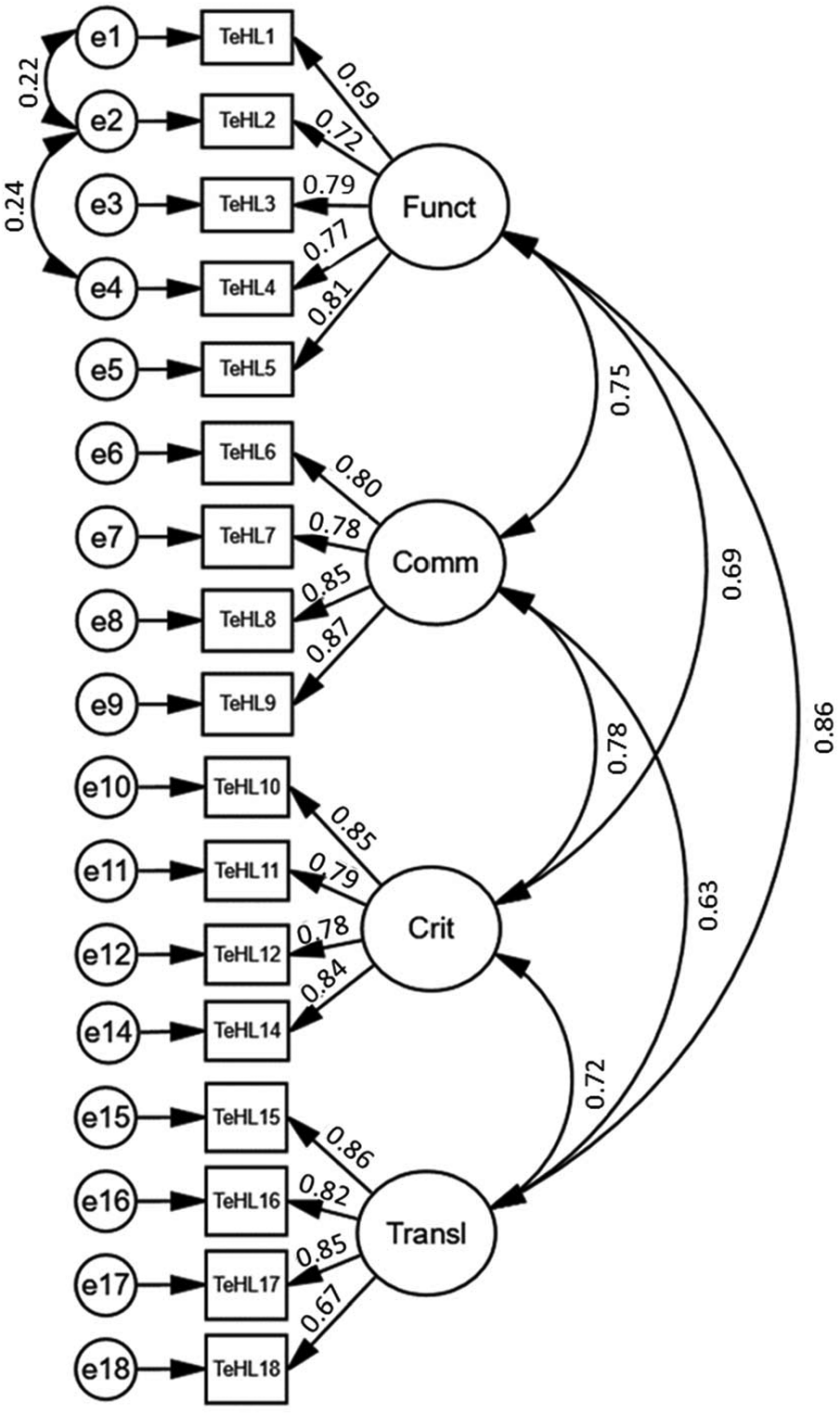
The four-factor measurement model for the Pl-TeHLI obtained after the exclusion of item 13 (TeHL1-18, items of transactional e-Health literacy instrument; Funct, “Functional” factor; Comm, “Communicative” factor; Crit, “Critical” factor; Transl, “Translational” factor).

The CFA showed that the four-factor model, after removing item 13 and including item 5 in factor 1 (“Functional”), best fits the measurement data. This four-factor measurement model for the Pl-TeHLI is shown in Figure 1. The fit indices for the model were as follows: CMIN = 4.697, NFI = 0.950, GFI = 0.932, AGFI = 0.907, CFI = 0.960, RFI = 0.938, TLI = 0.951, and RMESEA (90% CI) = 0.065 (0.060–0.071) (Table 3). An acceptable fit was also confirmed for the four-factor model, with all items preserved as in the original English version developed by Paige et al. (20).

The three-factor model, after the modification suggested by the results of the EFA, could also be fitted to measurement data on an acceptable level; however, all fit indices were less favorable for this model than for the four-factor models. We decided that the four-factor model we obtained with the EFA with an enforced number of factors should have priority as it adheres closely to the theoretical justification provided by the model of TeHL.

### 3.5 Reliability assessment

The reliability assessment was carried out for the TeHL subcategories based on the four-factor model of the instrument with the best fit to measurement data. The Cronbach *α* and Guttman half-split coefficients are presented in Table 4. They indicate very good internal consistency of all four subscales. The table also contains stability indicators—two-week mean and single-item ICCs confirming good or excellent stability of the subscales. Finally, floor and ceiling effects were calculated for all TeHL subcategories; they remained at an acceptable level. The floor effect was 0.4–1.9, and the ceiling effect was 3.2–7.3 (Table 4).

**Table 4:** Cronbach α and Guttman half-split coefficients, single item and mean ICC and floor and ceiling effect for the four factors distinguished in the TeHLI model.

TeHL subcategory	Functional	Communicative	Critical	Translational
Cronbach α coefficient	0.892	0.903	0.896	0.880
Guttman half-split coefficient	0.864	0.893	0.881	0.854
single item ICC (95% CI)	0.76 (0.68–0.82)	0.66 (0.56–0.74)	0.63 (0.52–0.72)	0.74 (0.65–0.80)
mean ICC (95% CI)	0.86 (0.81–0.90)	080 (0.72–0.85)	0.77 (0.69–0.84)	0.85 (0.79–0.89)
floor effect (%)	0.6	1.9	1.1	0.4
ceiling effect (%)	5.5	3.7	3.2	7.3

ICC, interclass correlation coefficient.

### 3.6 External consistency

The factors distinguished in the four-factor model of the TeHL instrument were moderately positively correlated (the Spearman rho coefficient ranged from 0.526 to 0.688. We have also checked the correlation of the subscores with the eHL score based on the eHEALS (Table 5). The eHEALS score was moderately positively correlated with all four TeHL subcategories. The correlation coefficient ranged from 0.568 to 0.642 (Table 5).

**Table 5:** TeHLI subcategories and eHEALS score correlations.

Subcategories of TeHLI	eHEALS	Functional	Communicative	Critical
Functional	0.653			
Communicative	0.568	0.640		
Critical	0.642	0.584	0.670	
Translational	0.630	0.688	0.526	0.591

*p*-value for all Spearman rho correlations <0.001.

### 3.7 The relationship between the categories of TeHL and the use of e-health services

In the logistic regression models developed for the use of six e-health services, we have applied independent variables corresponding with scores based on the four subscales distinguished in the TeHL instrument. Dichotomous dependent variables were based on the questionnaire's items asking about the use of services in the preceding 12 months. The frequencies of the use of e-health services are shown in Table 6. The use of e-health services ranged from 48.3% for the Internet Patient Account Portal to 6.1% for portals offering paid medical advice. The associations of the scores originating from the TeHL subcategories with dependent variables were adjusted for the sociodemographic characteristics of respondents.

**Table 6:** The use of e-health services by survey participants.

e-health service	*n*	%
Remote physician advice (telephone- or VTC-based)	604	45.9%^a^
Internet Patient Account Portal (IPAP)	803	48.3%
Portals providing health-related content	686	41.3%
Portals providing paid health advice	102	6.1%
Websites with test results	725	43.6%
Websites offering e-prescriptions	169	10.2%

^a^ The use of remote physician advice was assessed only among those who confirmed that they had needed physician advice in the preceding 12 months (*n* = 1,315).

Regression modeling revealed that both Functional eHL and Critical eHL showed a significant relationship with four, Translational eHL with three, and Communicative eHL with only two out of the six analyzed e-health services (Table 7). Respondents with greater Functional eHL were more likely to use remote physician advice by telephone or a videoconferencing system (OR, 95% CI: 1.51, 1.12–2.03), the Internet Patient Account Portal (OR, 95% CI: 1.33 (1.02–1.72), portals providing general health-related information (OR, 95% CI: 2.30, 1.72–3.08), and laboratory test results websites (OR, 95% CI: 1.68, 1.28–2.20). Interestingly, higher Critical eHL increased the likelihood of using portals offering paid medical advice (OR, 95% CI: 1.73, 1.10–2.72) and websites offering e-prescriptions (OR, 95% CI: 1.42, 1.01–2.00) but decreased the likelihood of utilizing remote physician advice available in the public healthcare system (OR, 95% CI: 0.76, 0.60–0.96) or websites allowing for checking laboratory test results (OR, 95% CI: 0.74, 0.60–0.92).

**Table 7:** The associations of the four types of TeHL with the use of e-health services assessed with multivariable logistic regression after adjusting for socio-demographic factors.

Dependent variable	Functional eHL		Communicative eHL		Critical eHL score		Translational eHL score	
	OR (95% CI)	*p*	OR (95% CI)	*p*	OR (95% CI)	*p*	OR (95% CI)	*p*
Remote physician advice (telephone- or VTC-based)	1.51 (1.12–2.03)	0.006	1.12 (0.91–1.37)	0.288	0.76 (0.60–0.96)	0.020	1.12 (0.85–1.49)	0.422
Internet Patient Account Portal (IPAP)	1.33 (1.02–1.72)	0.033	0.90 (0.75–1.09)	0.285	0.91 (0.74–1.11)	0.361	1.43 (1.12–1.84)	0.005
Portals providing health-related content	2.30 (1.72–3.08)	<0.001	0.76 (0.62–0.93)	0.007	0.93 (0.75–1.15)	0.479	2.33 (1.77–3.08)	<0.001
Portals providing paid health advice	1.19 (0.63–2.24)	0.590	1.75 (1.14–2.68)	0.010	1.73 (1.10–2.72)	0.018	0.94 (0.51–1.73)	0.835
Websites with test results	1.68 (1.28–2.20)	<0.001	0.97 (0.80–1.17)	0.746	0.74 (0.60–0.92)	0.006	1.36 (1.05–1.76)	0.019
Websites offering e-prescriptions	1.35 (0.85–2.13)	0.198	1.28 (0.94–1.75)	0.122	1.42 (1.01–2.00)	0.045	0.87 (0.56–1.36)	0.547

Respondents with higher Communicative eHL were more prone to use the paid medical advice portals (OR, 95% CI: 1.75, 1.14–2.68) but less likely to use portals providing general health-related information. Finally, higher Translational eHL was significantly associated with a higher likelihood of accessing the Internet Patient Account Portal (OR, 95% CI: 1.43, 1.12–1.84), general health information portals (OR, 95% CI: 2.33, 1.77–3.08), and websites allowing for checking laboratory test results (OR, 95% CI: 1.36, 1.05–1.76).

## 4 Discussion

### 4.1 Cultural adaptation and validation of the Polish version of TeHL instrument

We have conducted a cultural adaptation of the English version of the TeHL instrument to Polish, following the WHO guidelines (29). After cognitive interviewing, we have modified several items to improve their understanding by representatives of the general population. Then, we used the Polish version of the TeHL instrument in the survey conducted among a representative sample of adult Internet users to validate the instrument. The data set obtained from the survey was randomly split into two subsets. The first subset was used to perform an EFA based on the maximum likelihood method and the technique of oblimin rotation. Two variants of the EFA were implemented: first, with an enforced four-factor structure of the instrument, following the findings of the team that developed the original English version of the TeHL instrument [Paige et al. (20)], and second, allowing for the automatic establishment of the factor model. The CFA carried out on the first subset of data showed that the modified four-factor model obtained after exlcuding one item best fit the measurement data.

Paige et al. validated the TeHL instrument among a random sample of patients recruited from a university-based research registry (20). The CFA confirmed an acceptable fit of the four-factor model. Interestingly, the same team extended the TeHLI, adding a Clinical eHL subscale with 5 items (38). Acceptable fit was also confirmed for this extended version of TeHL instrument.

To date, only a few adaptations to other languages of the original English version of the TeHL instrument have been undertaken. We have identified only a few papers describing the validation of a culturally adapted version of the TeHL instrument or at least mentioning the use of this tool in the study (39–41). Nguyen et al. reported the results of the validation of the Vietnamese version of the TeHL instrument among a group of young adults (41). The authors performed the EFA for four models of TeHL instrument, with one, two, three, and four-factor structures. The four-factor model corresponded with the model established for the original English version of the instrument. It was confirmed that the four-factor model fits the measurement data best out of all four models.

Marzo et al. described the use of the TeHL instrument in the analysis of factors influencing parents' hesitancy to vaccinate their children against COVID-19 in Malaysia (40). Unfortunately, the paper did not provide information about the adaptation and the validity of the tool. The authors reported that only the “Communicative” component of TeHL had a significant relationship with vaccine hesitancy in parents in the univariable regression model. The TMeHL was also used by Kamaruzaman & Mohamad to guide the development of a tool used later in a qualitative study analyzing COVID-19 information-seeking behavior (Kamaruzaman & Mohamad, 2023).

Paige et al. showed that all four components of the TeHLI were at least moderately positively correlated with the eHEALS score (20). In our study, we have confirmed that four components of the Polish version of TeHLI are also significantly positively correlated with the eHEALS score; Spearman rho coefficients ranged from 0.526 to 0.688.

### 4.2 Transactional eHL and the use of e-health services

Our study revealed that at least 40% of respondents had used the Internet Patient Account Porta l in the preceding year, remote physician advice (either telephone- or VTC-based), an LTR website, and a general health information portal (48.3%, 45.9%, 43.6%, and 41.3%, respectively). A relatively high number (10.2%) of participants confirmed the use of EPR websites after online or telephone contact (without the need to make an appointment with a family physician's office that provides care within universal health coverage). Only 6.1% of the respondents had used paid medical advice portals.

Regression modeling of variables reflecting the use of e-health services yielded a rather complex image. A striking feature is the lack of a consistent pattern of relationships between the use of various types of e-health services and the four subcategories of eHL.

Functional eHL was a positive predictor of the use of four out of six e-health services (remote physician advice, the the Internet Patient Account Portal, general health information portals, and websites allowing for checking laboratory test results). All these services are either provided to all citizens with general health insurance or are free, like many portals offering health-related content. Online access to test results is commonly enabled to authorized users without additional payment by healthcare providers or facilities performing laboratory tests.

Functional eHL was not significantly associated with the use of paid medical advice portals or websites offering e-prescriptions. The latter services (sometimes called “virtual clinics”) became highly popular after the introduction of e-prescriptions and became a subject of significant controversy (42). Some doctors working for the providers of such services generated many thousands of prescriptions over the course of a year, which precluded reliable assessment of patient's health status and potentially could be a source of health risks (43). In many cases, such portals issued e-prescriptions based on a questionnaire filled out by a patient online outside the public healthcare system. The Ethical Commission of the Supreme Medical Chamber emphasized that online e-prescribing without adequate contact with a patient violates ethical rules that are obligatory for physicians [Ethical Commission of the Supreme Medical Chamber (44)]. According to the Supreme Medical Chamber, the online generation of e-prescriptions cannot be interpreted as remote physician advice accompanied by the issuing of an e-prescription. In our study, about 10% of respondents confirmed that they had utilized such portals to obtain e-prescriptions.

A significant positive relationship with the use of e-health services available freely to all citizens or at least to persons with general health coverage, like in the case of the Internet Patient Account Portal, may indicate that people with higher Functional eHL have greater knowledge and skills necessary to use the resources of the public healthcare system. They are also able to receive adequate support from such services and are not prone to turn to paid medical advice available online outside the public healthcare system. In the case of portals offering e-prescriptions after superficial online contact, higher Functional eHL may be related to better awareness of the potential risks of such a service.

Surprisingly, Communicative eHL had a limited impact on the use of e-health services. It was a significant predictor of only two out of the six analyzed services. The respondents with higher Communicative eHL were less likely to use general health information portals but more likely to utilize paid medical advice websites. The latter relationship may be explained by the fact that greater communication skills may be a factor that encourages the use of paid advice. The use of such a service requires the ability to communicate well, combined with the ability to use digital tools. Why Communicative eHL is adversely associated with the use of general health-related portals remains unclear. Such portals usually provide some means for communication with other users or even health professionals who offer general information about medical problems.

Critical eHL was significantly associated with the use of four out of six services, including remote physician advice, laboratory test results websites, paid medical advice portals, and finally, websites offering e-prescriptions. However, in the case of the first two services, it was a negative predictor. It may be surprising that persons with higher critical eHL are less prone to exploit remote physician advice by phone or videoconferencing system. It is possible that the individual characteristics that contribute to higher critical eHL result in lower trust in such a mode of receiving medical advice. It seems that Critical eHL may be perceived to some extent as a measure of lower trust toward routine services available in public healthcare.

On the other hand, higher Critical eHL predisposed respondents to the use of paid services, either in the form of medical advice or a “virtual clinic” issuing e-prescriptions after online contact. It is not clear what the mechanism is that is responsible for a higher preference for paid services and lower trust in public healthcare services. Maybe a critical attitude toward health-related resources is a by-product of the general critical assessment of the support offered by public healthcare systems. As a result, the fact that somebody pays additionally for the service, even online, is perceived by persons with highly critical attitudes as a guarantee of the quality or reliability of the service. On the other side, one could expect that persons with higher Critical eHL would be less eager to use online generators of e-prescriptions, especially after many warnings about the potential risks related to such services available in the media (42, 45).

We also observed that Translational eHL was a significant positive predictor of the use of the Internet Patient Account Portal, general health information portals, and websites allowing for checking laboratory test results. This finding supports the assumption that people are able to benefit from available e-health resources to the highest degree, either those provided by the state, as in the case of the the Internet Patient Account Portal, or by other entities active in the online content market to make decisions about health issues.

Based on the observed relationships between types of eHL and the use of specific e-health services, we can see that the associations of Translational eHL with dependent variables are parallel to these observed in the case of Functional eHL. To some extent, a similar observation is valid for Communicative and Critical eHL. The effects of these pairs of TeHL subcategories tend to be opposite regarding the use of the selected e-health services, or at least, a significant effect is observed on other sets of services. Our findings also tend to show that the interpretation of the meaning of the subcategories distinguished in the Model of TeHL should be more extensively researched. We observed a significant moderately positive correlation between the eHEALS score and TeHL subcategories and between pairs of TeHL subcategories; however, the associations revealed by regression modeling indicate that the meaning of the TeHL instrumentcomponents should need further explanation. This observation also supports the utilization of separate subscores generated for each TeHL subcategory in assessing the determinants of e-health service use rather than a combined score.

### 4.3 Limitations

We have conducted a cultural adaptation of the TeHL instrument and then validated the instrument on data originating from a survey among a large, extensive sample of adult Internet users. To assure methodological rigor, two subsets of data were generated by means of random splitting of the original data set. The fit of models generated with the EFA was then assessed with a CFA performed on another data set. However, we must admit that to preserve the four-factor model of the instrument, adhering to the theoretical model of TeHL proposed by Paige et al. (20), we enforced the analysis with a set number of factors, also relaxing, in this case, Kaiser's criterion for eigenvalues. We believe that the theoretical justification of the factor structure is permissible. A competing three-factor model obtained with the EFA without an enforced number of factors also showed an acceptable fit in the CFA but lower than the enforced four-factor model. Furthermore, the three-factor structure significantly distorted assumptions included in the Transactional Model.

So far, only a few teams have undertaken the effort to prepare a national version of the instrument for measuring TeHL. Such a situation limits the comparison of the influence of the resulting subscores on the use of e-health services. Available evidence showed that the subcategories of TeHL are significantly positively correlated with the score generated with the eHEALS. Our observations of the opposite effects of some components of the Model of TeH on the use of selected e-health services tend to show a rather complex mechanism of influence.

## 5 Conclusions

The introduction of the Polish version of TeHL instrument, consisting from 17 items, opens the way to research on the role of digital health literacy, going beyond the use of the eHEALS tool. The use of the four subscales encompassed in the Model of TeHL makes it possible to obtain a more nuanced vision of the determinants of the use of e-health services. The models developed in this study showed that components of e-health literacy may have opposite relationships with the use of services. Furthermore, each type of e-health service should be analyzed individually to understand the circumstances of their usage in the population.

## Data Availability

The datasets presented in this study can be found in online repositories. The names of the repository/repositories and accession number(s) can be found below: Zenodo https://zenodo.org/records/12594470.
