# Supplementary material for: Transactional e-health literacy and its association with e-health services use in Polish adults: a cross-sectional study
Source: Front Digit Health. 2024 Nov 22;6:1458650. doi: 10.3389/fdgth.2024.1458650 (PMC11621100; doi:10.3389/fdgth.2024.1458650)
Supplement: Supplementary file 1 [file Datasheet1.pdf]

Supplementary Material

Supplementary Material Table 1. English and Polish versions of Transactional eHealth Literacy Instrument.

Item	Transactional eHealth Literacy Instrument	Kwestionariusz Transakcyjnych Kompetencji e-Zdrowotnych
**1**	I can summarize basic health information from the Internet in my own words.	Potrafię podsumować własnymi słowami podstawowe informacje zdrowotne z Internetu, np. dotyczące zdrowego odżywiania lub aktywności fizycznej
**2**	I know how to access basic health information on the Internet.	Wiem, gdzie w Internecie mogę znaleźć podstawowe informacje o zdrowiu
**3**	I can use my computer to create messages that describe my health needs.	Potrafię przy użyciu komputera tworzyć wiadomości opisujące moje potrzeby zdrowotne, np. dotyczące badań profilaktycznych lub stylu życia.
**4**	I have the skills I need to tell someone how to find basic health information on the Internet.	Wiem, jak komuś pomóc znaleźć w Internecie podstawowe informacje o zdrowiu, np. dotyczące zdrowego odżywiania lub aktywności fizycznej
**5**	I can achieve my health information goals on the Internet while helping other users achieve theirs.	Potrafię korzystać z informacji zdrowotnych w Internecie, jednocześnie pomagając innym internautom w korzystaniu z takich informacji
**6**	I have the skills I need to talk about health topics on the Internet with multiple users at the same time.	Mam umiejętności potrzebne do prowadzenia w Internecie rozmów na tematy zdrowotne z wieloma użytkownikami jednocześnie
**7**	I can identify the emotional tone of a health conversation on the Internet.	Potrafię rozpoznać emocjonalny ton rozmowy w Internecie na temat zdrowia
**8**	I have the skills I need to contribute to health conversations on the Internet.	Mam umiejętności potrzebne, aby włączyć się w Internecie do dyskusji dotyczącej zdrowia
**9**	I have the skills I need to build personal connections with other Internet users who share health information. c	Wiem, jak utrzymywać relacje z innymi użytkownikami Internetu, którzy dzielą się informacjami o zdrowiu
**10**	I can tell when an Internet user is a credible source of health information.	Potrafię rozpoznać, czy użytkownik Internetu jest wiarygodnym źródłem informacji zdrowotnych
**11**	I can tell when health information on the Internet is fake.	Potrafię stwierdzić, kiedy informacje zdrowotne w Internecie są nieprawdziwe
**12**	I can tell when a health website is safe for sharing my personal health information.	Potrafię stwierdzić, czy strona internetowa dotycząca zdrowia jest bezpieczna, aby podzielić się na niej osobistymi informacjami zdrowotnymi

**13**	I can tell when information on the Internet is relevant to my health needs.	Potrafię określić, które informacje zdrowotne w Internecie są dla mnie przydatne
**14**	I know how to evaluate the credibility of Internet users who share health information.	Wiem, jak ocenić wiarygodność użytkowników Internetu, którzy dzielą się informacjami zdrowotnymi
**15**	I can use the Internet to learn how to manage my health in a positive way.	Potrafię posłużyć się Internetem, aby nauczyć się, jak zadbać o swoje zdrowie
**16**	I can use the Internet as a tool to improve my health.	Potrafię wykorzystać Internet jako narzędzie do poprawy swojego zdrowia
**17**	I can use information on the Internet to make an informed decision about my health.	Potrafię korzystać z informacji w Internecie w taki sposób, aby podejmować świadome decyzje o swoim zdrowiu
**18**	I can use the Internet to learn about topics that are relevant to me.	Potrafię skorzystać z Internetu do zdobywania wiedzy na ważne dla mnie tematy

Supplementary Material Table 2. Total variance explained by the four-factor latent structure of the Polish version of Transactional e-Health Literacy Instrument

Factor	Initial Eigenvalues			Sum of Squared Loading after Extraction			Sums of squared loadings after rotation
	% of variance	Cumulated % of variance	Total	% of variance	Cumulated % of variance	Total	
**1**	10.223	56.793	56.793	9.920	55.113	55.113	7.929
**2**	1.560	8.669	65.462	1.257	6.982	62.095	5.658
**3**	1.142	6.346	71.808	0.850	4.724	66.819	7.509
**4**	0.759	4.218	76.026	0.482	2.676	69.496	7.416
**5**	0.542	3.010	79.036				
**6**	0.504	2.800	81.836				
**7**	0.407	2.263	84.099				
**8**	0.361	2.006	86.105				
**9**	0.352	1.954	88.059				
**10**	0.322	1.789	89.848				
**11**	0.293	1.627	91.475				
**12**	0.281	1.563	93.038				
**13**	0.263	1.460	94.498				
**14**	0.225	1.252	95.75				
**15**	0.219	1.218	96.968				
**16**	0.207	1.152	98.12				
**17**	0.176	0.977	99.096				
**18**	0.163	0.904	100				

Supplementary Material Table 3. Pattern matrix resulting from exploratory factor analysis performed with Maximum Likelihood method and direct oblimin rotation.

Item	Factor 1	Factor 2	Factor 3	Factor 4
**1**	0.549	-0.073	0.041	-0.099
**2**	0.794	0.156	0.070	-0.114
**3**	0.679	-0.165	0.027	-0.030
**4**	0.833	0.031	0.019	-0.075
**5**	0.760	-0.28	-0.011	0.100
**6**	0.132	-0.729	0.044	-0.035
**7**	0.113	-0.508	0.25	-0.113
**8**	0.058	-0.737	0.059	-0.151
**9**	0.112	-0.615	0.202	-0.066
**10**	-0.063	-0.182	0.771	-0.023
**11**	0.023	0.04	0.952	0.100
**12**	0.063	-0.019	0.711	-0.054
**13**	0.191	0.028	0.320	-0.377
**14**	-0.002	0.001	0.758	-0.114
**15**	-0.051	-0.084	0.055	-0.838
**16**	-0.033	-0.17	-0.034	-0.813
**17**	0.152	-0.013	0.051	-0.703
**18**	0.16	0.133	0.025	-0.608
